# LDF-BNN: A Real-Time and High-Accuracy Binary Neural Network Accelerator Based on the Improved BNext

**DOI:** 10.3390/mi15101265

**Published:** 2024-10-17

**Authors:** Rui Wan, Rui Cen, Dezheng Zhang, Dong Wang

**Affiliations:** 1 Institute of Information Science, Beijing Jiaotong University, Beijing 100044, China; 22110093@bjtu.edu.cn (R.W.); 22120306@bjtu.edu.cn (R.C.); dezhengzhang@bjtu.edu.cn (D.Z.); 2 Beijing Key Laboratory of Advanced Information Science and Network Technology, Beijing 100044, China

**Keywords:** binary neural networks, FPGA, high-accuracy, hardware accelerator

## Abstract

Significant progress has been made in industrial defect detection due to the powerful feature extraction capabilities of deep neural networks (DNNs). However, the high computational cost and memory requirement of DNNs pose a great challenge to the deployment of industrial edge-side devices. Although traditional binary neural networks (BNNs) have the advantages of small storage space requirements, high parallel computing capability, and low power consumption, the problem of significant accuracy degradation cannot be ignored. To tackle these challenges, this paper constructs a BNN with layered data fusion mechanism (LDF-BNN) based on BNext. By introducing the above mechanism, it strives to minimize the bandwidth pressure while reducing the loss of accuracy. Furthermore, we have designed an efficient hardware accelerator architecture based on this mechanism, enhancing the performance of high-accuracy BNN models with complex network structures. Additionally, the introduction of multi-storage parallelism alleviates the limitations imposed by the internal transfer rate, thus improving the overall computational efficiency. The experimental results show that our proposed LDF-BNN outperforms other methods in the comprehensive comparison, achieving a high accuracy of 72.23%, an image processing rate of 72.6 frames per second (FPS), and 1826 giga operations per second (GOPs) on the ImageNet dataset. Meanwhile, LDF-BNN can also be well applied to defect detection dataset Mixed WM-38, achieving a high accuracy of 98.70%.

## 1. Introduction

Industrial defect detection plays a vital role in industrial production. It can adjust the production process in time, reduce the rate of defective products, improve the overall production efficiency, and effectively reduce the production cost. Deep neural networks (DNN) are widely used for defect detection in steel [1], semiconductors [2], textiles [3] and other fields. This is because they are able to automatically learn and accurately identify different types of defects, minimizing the interference of human factors.

However, DNNs have the disadvantages of a large number of parameters and high computational complexity. To address these limitations, researchers have turned to binary neural networks (BNNs). BNNs have the advantages of accelerating computing speed, reducing storage requirements, lowering energy consumption, and enhancing model robustness [4,5,6], which make them widely applicable in industrial production.

Therefore, high-performance BNN accelerators based on Field-Programmable Gate Array (FPGA) devices are attracting increasing attention, offering superb power efficiency and design flexibility, and generating a wealth of research [4,5,6]. Among these, FINN [4] is an end-to-end framework for design space exploration and the creation of fully customized hardware on FPGAs, though it is limited by its low accuracy. Nakahara et al. [5] used custom JPEG encoding to compress transported images, accelerating image transmission to achieve excellent frame rate and throughput, yet this method faced restrictions revolving around leveraging 8-bit activation values to increase accuracy and the need for substantial hardware resources. FracBNN [6] achieved higher post-quantization accuracy by first quantizing the activation to 2-bit and then proceeding with sparsity. However, this also increased the usage of Block RAM (BRAM) resources. Currently, the BNN architectures utilized in accelerators on FPGA device tend to embody a level of simplicity. Hence, their accuracy is too low to be applicable in industrial inspection.

Recently, BNext [7] outperformed previous BNNs [8,9] to achieve accuracy comparable to DNNs. Song et al. [10] deployed the Bi-RealNet model on a hardware platform with scarce hardware resources. However, the accuracy has not yet reached the current optimum level, and the rate is merely 24 frames per second (FPS). Ma et al. [11] used a quantized RPReLU based on the ReActNet model to improve the accuracy to some extent while reducing the consumption of hardware resources. However, the accuracy has not yet reached the current optimum level. BNext [7] has significantly raised the accuracy boundary of the BNNs to 80.57%, making it comparable to CNN models like ResNet-101 and ResNet-50. Nevertheless, BNext is not hardware-friendly because of complex structures (feed-forward and feedback branches) and intricate network layers (such as 1 × 1 convolution, pooling, sigmoid, etc.). The complex structures can impose pressure on the bandwidth, and accelerating quantity of 1 × 1 convolutions requires the acceleration model to have a high transmission rate for input feature maps. In order to surmount the constraints of previous research, we put forward an efficient hardware accelerator based on the BNN with layered data fusion mechanism (LDF-BNN). The contributions of this work can be generalized as follows:

(1) The LDF-BNN model based on BNext was proposed. The design of the high-accuracy BNext model does not take into account the data streaming requirements of actual hardware deployment, resulting in bandwidth constraints and an overall model computational efficiency of only 38.18%. Based on this, we introduce a layered data fusion mechanism (LDF). By fusing data from different layers, LDF significantly reduces bandwidth requirements by 53.67%. Importantly, the loss of accuracy is almost negligible.
(2) An efficient hardware architecture based on LDF-BNN is devised. Given the relatively complex hardware architecture required to adapt LDF-BNN, and the fact that there is no general-purpose hardware accelerator available for deployment, we design this architecture. The approach achieves pipelining of LDF-BNN modules and improves the overall computational efficiency by 31.52% and by 1.83× speedup.
(3) An innovative multi-storage parallelism (MSP) design has been introduced. Even after model structure optimization, there exists a bottleneck in the rate at which convolution calculations read feature map data, resulting in the efficiency of 1×1 convolution remaining only 31.68%. The multilevel buffers of the aforementioned hardware architecture require more multi-dimensional parallelism to meet the demand for reading rates. Hence, MSP is proposed. This design, in turn, partially improves the overall model computational efficiency by 14.75%, and achieves a 2.21× speedup.
(4) This design is fully implemented on the Xilinx ZCU102 platform. Experimental results indicate that the proposed LDF-BNN accelerator attains a high accuracy of 72.23%, an image processing rate of 72.6 FPS and 1826 GOPs on the ImageNet dataset when the system clock frequency is set at 200 MHz. Our design also maintains a high accuracy of 98.70% on the Mixed WM-38 dataset [12], which can demonstrate the potential of LDF-BNN in the field of defect detection.

## 2. Proposed BNN Architecture

### 2.1. Bottlenecks in Previous Design

BNext introduces a methodology incorporating an adaptive information recoupling structure and element-wise attention mechanism into binary convolutional networks. This approach is distinguished by its capability to enhance the correlation between signals before and after binary convolution, presenting a smoother loss profile compared to prevailing designs. This characteristic facilitates ease of optimization. While demonstrating clear advantages in model training, this methodology is confronted with the following challenges.

**Figure 1:**
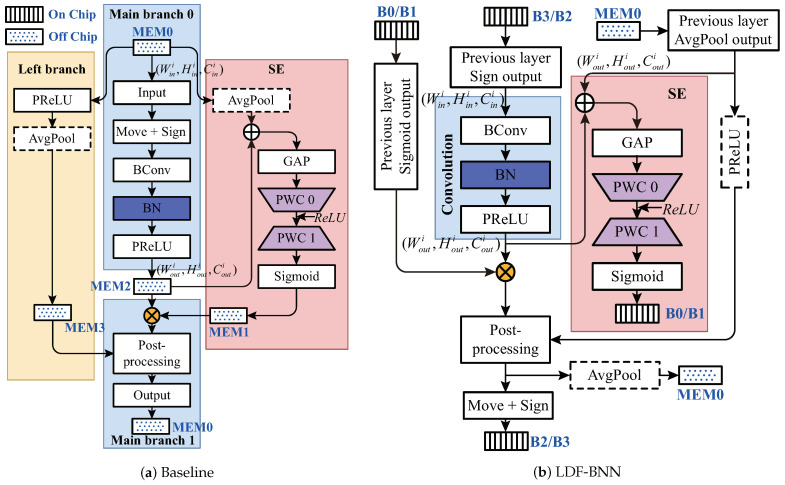
The single-layer structure. The meanings of the modules BConv, BN, GAP, PReLU, PWC, and Move can all be referred to in Appendix A.

The single-layer structure of BNext-Small [7] with the complex structures is depicted in Figure 1a. We can set the methodology, where the weights/inputs/outputs of the convolution part are accessed exactly once per each layer from the off-chip memory, as the baseline for further examination. Moreover, constrain the data transfer between the left branch, SE, the main branch 0, and the main branch 1 on the baseline to merely access through off-chip dynamic random access memory (DRAM). The main branch 0 contains [Sign-BConv-BN-PReLU] operations. The left branch includes [PReLU-AvgPool] operations. SE involves operations of merging inputs and outputs of main branch 0, followed by [GAP-PWC 0-PWC 1]. Main branch 1 contains a post-processing operation that first multiplies the outputs of main branch 0 and SE, conducts BN, and then combines it with the output of the left branch. In this architecture, the data, alongside main branch 0, generates a feedback channel after passing through PReLU. This implies that the subsequent computations in main branch 1 need to wait until the calculations in the right SE branch have completely concluded. Moreover, the intricate, multi-branch network structure necessitates frequent data read and write operations in DRAM.

**Figure 2:**
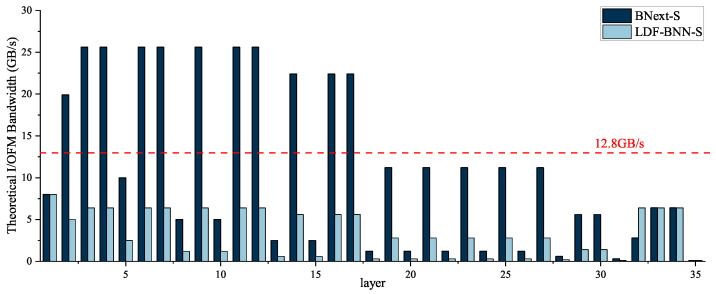
Comparison of theoretical I/OFM bandwidth.

Using the *i* layer as an example, assuming that all data transmitted to DRAM is quantized to 8-bit, the input size for Input is $\left(W_{in}^{i}\times H_{in}^{i}\times C_{in}^{i}\right)=S_{in}^{i}$, and the output size for BConv is $\left(W_{out}^{i}\times H_{out}^{i}\times C_{out}^{i}\right)=S_{out}^{i}$. Then, main branch 0 needs to read data of size $S_{in}^{i}$ bytes, and write data of size $S_{out}^{i}$ bytes. Similarly, the left branch needs to read data of size $S_{in}^{i}$ bytes, and write data of size $S_{out}^{i}$ bytes. SE needs to read data of size $\left(S_{in}^{i}+S_{out}^{i}\right)$ bytes, and write data of size $C_{out}^{i}$ bytes. Finally, main branch 1 needs to read data of size $\left(2S_{out}^{i}+C_{out}^{i}\right)$ bytes, and write data of size $S_{out}^{i}$ bytes. If the above baseline ignores the waiting time of the SE part and only considers the feature map mapping, then SE, left branch, and main branch 1 can all perform element-wise calculations with the output data of BConv. At the same time, the theoretical maximum bandwidth is $\left(3S_{in}^{i}+5S_{out}^{i}+2C_{out}^{i}\right)/t_{Bconv}^{i}$, where $t_{Bconv}^{i}$ represents the Bconv calculation time. Consequently, as depicted in Figure 2 the theoretical maximum bandwidth required for a single layer of BNext-Small would be 25.6 GB/s. This requirement exceeds the peak bandwidth of 12.8 GB/s provided by the Xilinx ZCU102 platform. As shown in Section 4.2, the overall computational efficiency of the model is only 38.18%. This low efficiency suggests that the specific design structure may not be suitable for hardware optimization.

### 2.2. Proposed LDF-BNN Architecture

Based on the bandwidth bottleneck analyzed above, we construct the LDF-BNN architecture to optimize the overall performance of the system.

LDF: To address the above issues, we advocate a novel approach and construct the LDF-BNN model by introducing a layered data fusion mechanism, which is shown in detail in Figure 1b. The LDF-BNN model structure can be divided into three parts: Convolution, SE, and Others. The SE section has been modified to a cross-layer structure, enabling the main branch to proceed with deep pipelining operations without waiting for SE, while the SE segment runs in parallel with the main branch. This mechanism avoids the limitation of single-level information and reduces the loss of accuracy by using the information of interval levels. It is worth noting that there is no information available from the previous layer in the $1^{\mathrm{st}}$ layer, so this article introduces $C_{out}^{1}$ cross-layer learnable parameters are trained to obtain optimal information.

Squeeze-and-excitation: The red area in Figure 1b is precisely the squeeze-and-excitation module. It serves as a key component that corrects feature maps by learning channel interdependencies. The module has two main parts: squeezing and excitation. In squeezing, GAP compresses spatial info into a single value per channel. In excitation, a two-layer neural network generates channel weights for channel multiplication operations to suppress minor channels. It is worth noting that the element-wise addition before GAP is also classified as SE for the convenience of hardware pipelining. Appendix A shows that using Sigmoid activation after PWC 1 is more competitive than using ReLU activation.

Dimensionality reduction operation pre-placement: In addition, we adopted a dimension reduction operation pre-placement strategy of swapping the positions of PReLU and AvgPool in the Left Branch of BNext. Since PReLU mainly operates on each channel, after adjustment, we can reduce the computational workload and hardware resources of AvgPool without sacrificing accuracy.

**Figure 3:**
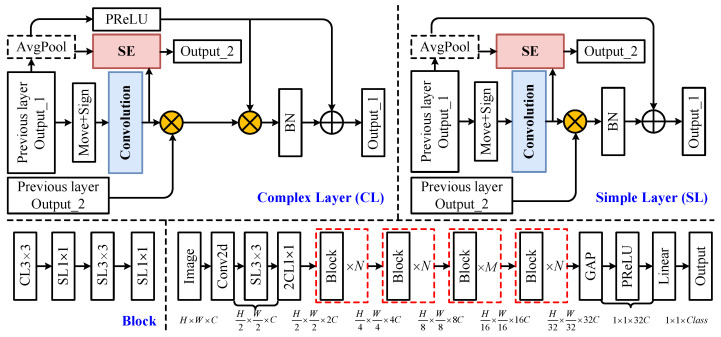
The overall architecture of LDF-BNN. “N” and “M” are the number of blocks. SL and CL, respectively, denote Simple Layer and Complex Layer.

Block design: We construct the complete LDF-BNN architecture by stacking blocks, and blocks are composed of two basic hierarchical structures: Complex Layer (CL) and Simple Layer (SL). The block shown in Figure 3 is entirely composed of [CL3×3-SL1×1-SL3×3-SL1×1]. It is worth noting that when the stride is 2, SL1×1 will be doubled and then combined with concat to increase the dimension. The AvgPool module introduced in a single hierarchical structure will ensure the consistency of the front and back sizes. In addition, we refer to BNext to configure LDF-BNN as shown in Table 1.

The above strategies can all reduce the number of DRAM read and write operations. As illustrated in the figure, the optimized model and data flow exist solely with SE reading $S_{out}^{i}$ bytes data, PReLU pulling $S_{out}^{i}$ bytes data from MEM0 and AvgPool recording $S_{out}^{i}$ bytes data. Relative to the baseline, there is a data read-write saving of $\left(2S_{in}^{i}+4S_{out}^{i}+2C_{out}^{i}\right)$ bytes, thereby liberating the bandwidth. Subsequently, we compared the DRAM data transfer volume for the weights and input/output feature maps(I/OFMs) of all layers in the BNext-S and LDF-BNN-S using the calculation method described in the previous paragraph, and the results are shown in Table 2. The tabulated data reveals that, in comparison with the baseline, the optimizations have led to a bandwidth requirement reduction of 53.67%. When paired with the hardware architecture described in Section 4.2, it can achieve a speedup of 1.83 times. Furthermore, the achieved accuracy is 75.8%, which is comparable to the baseline.

### 2.3. Model Quantification

Finally, we perform 8-bit quantization using the learning step size quantization (LSQ) method [13] on the weights/inputs/outputs of the PReLU/AvgPool/GAP/PWC/BN modules for all layers. For the convolution part, except for the weights/inputs of the first layer Conv2d and the last layer Linear and the outputs of all convolutional layers, which are also quantized to 8-bit, the weights/inputs of the Bconv of the remaining layers are quantized to 1-bit.

Module merging: The calculation of BN involves a large number of complex multiplication and division operations, which consumes a lot of computing resources. To simplify hardware resources, according to [14], during the quantization process, we merge the BN of the convolution in Figure 1b into BConv. At the same time, in the post-processing stage, BN is simplified to element-wise multiplication and addition operations.

(1) $$\begin{array}{c} y\leftarrow \gamma^{\prime }x+\beta^{\prime }\equiv {\mathrm{BN}}_{\gamma^{\prime },\beta^{\prime }}\left(x\right) \end{array}$$

(2) $$\begin{array}{c} y\leftarrow a^{\prime }\cdot \mathrm{MAC}\left(x,w\right)+b^{\prime }\equiv {\mathrm{Conv}}_{a^{\prime },b^{\prime }}\left(x\right) \end{array}$$

where $\gamma^{\prime }$ and $\beta^{\prime }$ represent the learnable scale and bias in BN, $a^{\prime }$ and $b^{\prime }$ represent the learnable scale and bias after convolution merge BN, and MAC represents the multiplication and accumulation operation.

Learnable GAP factor: GAP requires multiplication by $s_{GAP}$, where $s_{GAP}=1/\left(H^{i}\cdot W^{i}\right)$. If this factor is directly quantized to 8-bit, it will cause additional quantization errors when the factor value is small. To solve this problem, we optimize this factor to a learnable parameter with an initial value of $1/(H^{i}\cdot W^{i})$. As shown in Section 4.1, even if this parameter is quantized to 8-bit precision, the decrease in accuracy is small.

## 3. The Framework of Hardware

### 3.1. Overall Hardware Architecture

**Figure 4:**
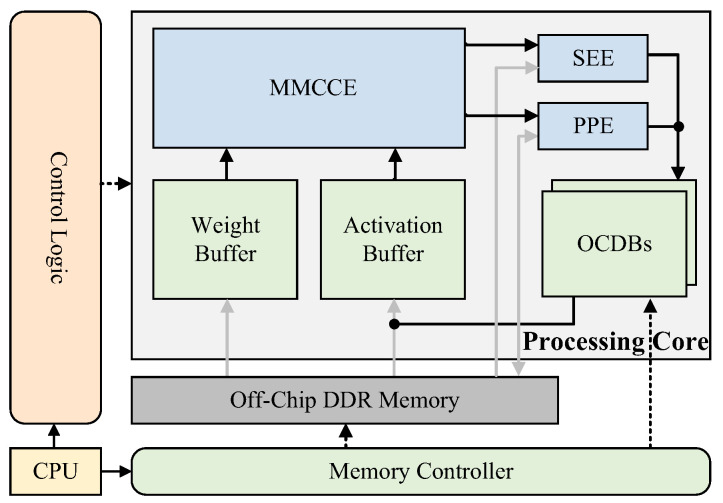
The overall architecture of the proposed accelerator for BNNs.

We have optimally utilized FPGA resources to design a high-performance BNN accelerator based on FPGA. As depicted in Figure 4, the overall architecture of the proposed accelerator for BNNs primarily incorporates a control logic, a memory controller, and a processing core. The proposed accelerator employs the parallelism inherent within the processing core to accomplish the BNN inference computational tasks. The control logic is instrumental in scheduling diverse hardware units for computation execution and overseeing data synchronization.

The processing core comprises the following functional modules: Multi-Mode Convolution Computation Engine (MMCCE), On-Chip Data Buffers (OCDBs), SE module Engine (SEE), and Post-Processing Engine (PPE). The convolution module in Figure 1b, which comprises the [BConv-BN-PReLU] computations, is mapped to the MMCCE for acceleration. The design of OCDBs acts as a ping-pong buffer to accommodate inter-layer data of the Sign and Sigmoid. The SEE module implements the point-wise convolution as the red segment shows in Figure 1b, which can execute independently along with the MMCCE and PPE modules such that inter-layer dependency are avoided. The remaining white regions in Figure 1b correspond to [PReLU-[fusion of the output from the previous layer’s Sigmoid and the convolution output]-Sign/AvgPool] computations for a single layer of the model, which is conducted by the PPE.

### 3.2. Multi-Mode Convolution Computation Engine

**Figure 5:**
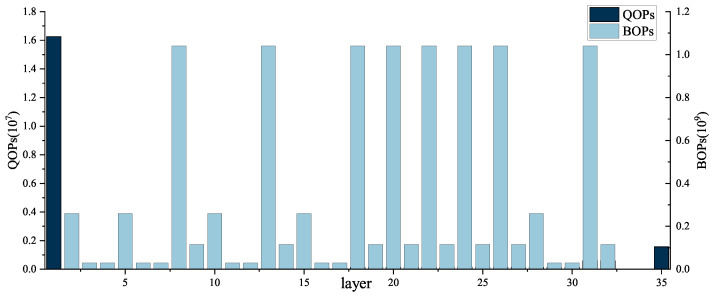
Comparison of BOPs and QOPs across all layers.

In Figure 5, we have accounted for the operations of 8-bit and 1-bit convolutions for each layer of the optimized BNext model, denoted as QOPs and BOPs, respectively. It is evident from the data that there is a substantial discrepancy between the quantities of QOPs and BOPs, with BOP being two orders of magnitude higher than QOP. Furthermore, QOP is predominantly concentrated in the first layer and in the final layer. In order to maximize the reuse of hardware resources between the two types of calculations, consequently improving the execution efficiency of the data path, we have designed a unified Multi-Mode Convolution Computation Engine (MMCCE) to implement both 1-bit and 8-bit convolution operation in one hardware module.

**Figure 6:**
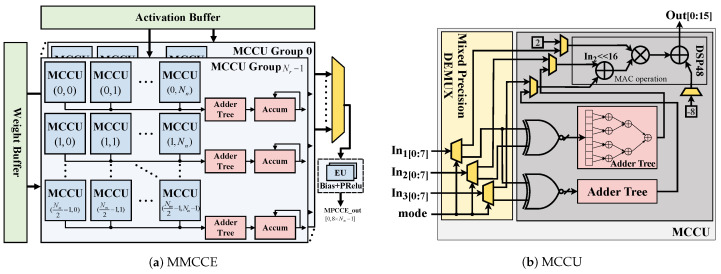
Structure of the Multi-Mode Convolution Computation Engines (MMCCEs). The yellow part represents the multiplexer (MUX) or demultiplexer (DEMUX). The Accum represents the accumulator.

As shown in Figure 6a, a crucial component of the MMCCE is the array of Minimal Convolution Computation Units (MCCUs). These units receive weights and activations through a streaming data influx from their respective buffers—the weight buffer and the activation buffer. Figure 6b further illustrates the exceptional versatility provided by the configurability of the MCCU within the MMCCE framework in supporting computations at two levels of precision. The MCCU can set different modes depending on the convolution precision of different layers. In 8-bit convolution mode, $In_{2}$ is first added to $In_{3}$, and the result is then multiplied by $In_{1}$; hence, a digital signal processing (DSP) can complete two 8-bit multiply accumulate(MAC) operations. In 1-bit convolution mode, $In_{2}$ and $In_{3}$ are first subjected to XNOR operations with $In_{1}$, and then their respective popcounts are performed by adder trees. The final data can be fed into the DSP for subsequent computations; hence, a DSP can complete sixteen 1-bit MAC operations.

**Figure 7:**
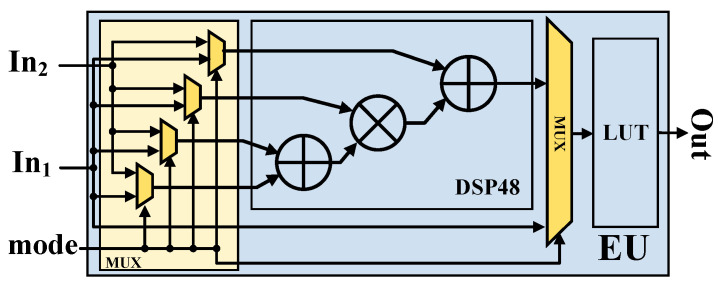
Structure of element-wise units (EUs).

As shown in Figure 6a, MMCCE adopts a multi-dimensional parallelism strategy to accelerate the convolutional layers of the network model. MMCCE includes $N_{r}$ MCCU Groups among its components. Each MCCU Group consists of $N_{m}/2$ rows and $N_{n}$ columns of MCCUs, as well as $N_{m}/2$ accumulation modules to compute the intermediate results of the convolution. The MMCCE collectively execute $N_{r}\times N_{m}\times N_{n}$ 8-bit MAC operations or $8\times N_{r}\times N_{m}\times N_{n}$ 1-bit MAC operations in each clock cycle. Note that we obtained the deviation and PReLU results by using the DSP of element-wise units (EUs) in Figure 7 and choosing between element-wise addition and element-wise addition with multiplication. Moreover, in this article, EUs can also execute element-wise sign and lookup-table operations by using the DSP of lookup tables (LUTs).

### 3.3. The Design of On-Chip Data Buffers

**Figure 8:**
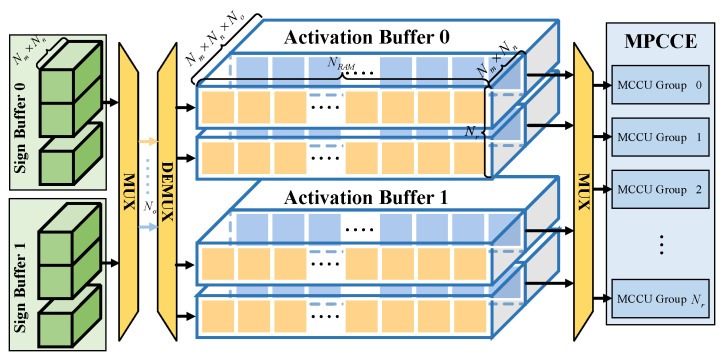
Multi-storage parallelism. The $N_{RAM}$ represents the preset size of RAM.

To accommodate MMCCE, this paper specifically designs a distinct Activation Buffer and Weight Buffers for the input feature map and weights, respectively. Following the LDF-BNN, we have devised a Sign Buffer for storing values that have undergone Sign operations on the chip with the objective of reducing the need to store values off-chip and thus alleviate bandwidth pressure. However, due to the high speed of Conv1×1 computations, the rate at which input feature map data are read becomes a bottleneck. In a situation where only the neural network has been optimized, and where $N_{n}=3$, $N_{m}=16$, and $N_{r}=14$, the overall efficiency of the Conv1×1 stands at merely 31.68%. The single block Activation Buffer of the baseline model, as illustrated in the yellow section of Figure 8, can store a total amount of data equal to $N_{m}\times N_{n}\times N_{RAM}\times N_{r}$. In each cycle, it reads $N_{m}\times N_{n}$ from the Sign Buffer.

To address the aforementioned issue, this work introduces a novel design of multi-storage parallelism (MSP) levels, as depicted in the blue section of Figure 8. An addition of $N_{o}$ is incorporated as the factor of on-chip storage parallelism. It amplifies the size of data transmitted in a single cycle by $N_{o}$ times. Such an improvement partially enhances the computational efficiency of the Conv1×1. In a setting where $N_{o}=2$, the computational efficiency of the Conv1×1 can be improved to 42.46%. Further details are discussed in Section 4.2.

Sigmoid Buffer is employed to store on-chip values after undergoing the Sigmoid operation. It utilizes a simple ping-pong caching mechanism. Additionally, the resources occupied by the Sigmoid Buffer on the chip are only the size of the maximum depth, which is 1536 × 2 bytes.

### 3.4. SE Module Engine

**Figure 9:**
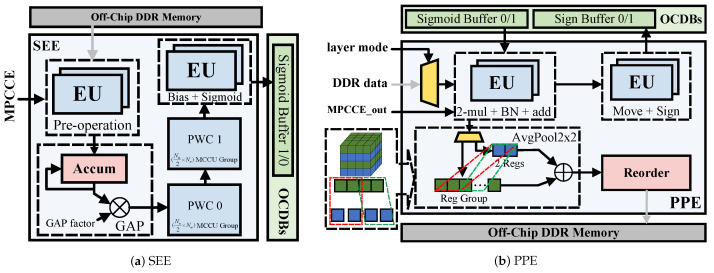
Structure of SE module Engine (SEE) and Post-Processing Engine (PPE).

The red area in Figure 1b corresponds to Figure 9a. The operation conducted by the pre-operations of SEE involves multiplication of the data retrieved from MEM0 and the data output from the blue area PReLU by their respective parameters, followed by addition. This process can be directly executed using EUs. Within the framework of this paper, the input/output results of the convolution, upon undergoing pre-operations, can participate in the pipeline processing of the GAP, thereby eliminating the need to wait until all the results are output before carrying out the accumulation process.

**Figure 10:**
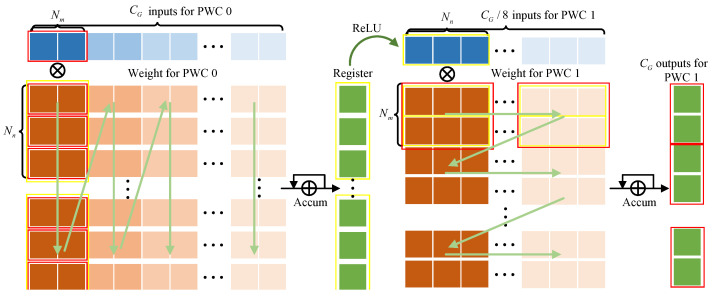
Calculation process for PWC 0 and PWC 1.

In PWC 0 and PWC 1, only one MCCU group is deployed for computation in each of them, since the dimension of the input feature map is 1. In order to overlap the calculation of PWC 0 and GAP, after PWC 0 obtains the $N_{m}$ parallel output results of GAP, we first multiply and accumulate the corresponding parts of all convolution kernels, and store the results in registers. According to Figure 10 and Algorithm 1, it can be seen that after $n_{n}$ clock cycles, the first $N_{m}$ columns of data are multiplied and summed to obtain $C_{G}/8$ results. The register is summed up again every $n_{n}$ clock cycles until $n_{n}\times n_{m}$ clock cycles. At this point, the values of the registers, after ReLU operations, become the inputs to PWC 1. The computation of PWC 1 is the same as a normal convolutional pipeline. For each $N_{m}$ result, just multiply and accumulate all $N_{m}$ rows of data without additional registers.
**Algorithm 1** PWC 0 in the SEE**Input:** The output results of the GAP $o_{G}$, GAP channel number $C_{G}$, PWC 0 output channel number $C_{G}/8$, the weights of PWC 0 w, Input/output parallelism $N_{m},N_{n}$. **Output:** $o_{P}$  1:$n_{m}=\mathrm{ceil}\left(C_{G}/N_{m}\right)$ 2:$n_{n}=\mathrm{ceil}\left(\left(C_{G}/8\right)/N_{n}\right)$ 3:**for** ii←0 **to** $n_{m}$ **do** 4:  **for** jj←0 **to** $n_{n}$ **do** 5:   **for** j←0 **to** $N_{n}$ **do** 6:    **#pragma unroll by factor** $N_{m}\cdot N_{n}$ 7:    **if** ii=0 **then** 8:     $\mathrm{reg}\left[jj\right]\left[j\right]=\sum_{i=0}^{N_{m}}o_{G}\left[ii\right]\left[i\right]\cdot w\left[jj\right]\left[j\right]\left[ii\right]\left[i\right]$ 9:    **else**10:     $\mathrm{reg}\left[jj\right]\left[j\right]+=\sum_{i=0}^{N_{m}}o_{G}\left[ii\right]\left[i\right]\cdot w\left[jj\right]\left[j\right]\left[ii\right]\left[i\right]$11:    **end if**12:   **end for**13:  **end for**14:**end for**15:$o_{P}=\mathrm{reg}$

It was observed that, after 8-bit quantization, the decimal point numbers of the fixed point inputs and outputs before and after the Sigmoid are concentrated approximately around 8 and 9. To exploit the observed concentration of values, we developed a lookup table approach. According to the different fixed-point positions of input and output, a lookup table of 4 $\times \;2^{8}$ bytes is obtained through permutations and combinations. Under the aforementioned parallelism, the method adopting a lookup table approach necessitated only 178 LUTs.

### 3.5. Post-Processing Engine

The remaining white area in Figure 1b is mapped to Figure 9b. BN, multiplication and addition of various branches, Move, and Sign are all calculated using EU. In addition, Figure 9b also demonstrates the calculation method of AvgPool. Assuming that *r* is the number of rows currently traversed, the register first stores the data of the ${\left(r-1\right)}^{th}$ row. For every two data inputs in the $r^{th}$ row, the corresponding data of the ${\left(r-1\right)}^{th}$ row is accumulated and shifted.

## 4. Experimental Results and Analysis

### 4.1. Software Performance Analysis

Both the full-precision model and the quantized model of LDF-BNN were trained on 2× NVIDIA Geforce RTX 4090 GPU and Intel i9-9900k CPU, and Pytorch version 1.13.1 was employed. The hyperparameters of the training phase for the full-precision model were configured as follows: the initial learning rate was set to $10^{-3}$, the minimum learning rate was $10^{-7}$, the number of training rounds was 512, the batch size was 128, the optimizer was AdamW [15], the weight decay rate was $10^{-8}$, and the scheduler was CosineLRScheduler. The quantization utilized the LSQ algorithm in quantization aware training (QAT). The hyperparameters of the training phase for the quantized model were set as follows: the initial learning rate was $10^{-6}$, the minimum learning rate was $10^{-7}$, the number of training rounds was 128, the batch size was 17, the optimizer was AdamW, and the scheduler was CosineLRScheduler.

#### 4.1.1. Comparison with Different BNNs

ImageNet: Table 3 shows the accuracy of the advanced BNNs for classification task on ImageNet. Our proposed model LDF-BNN is close to BNext, which is the current state-of-the-art (SOTA) design. At the same time, it is at least 1.7% higher than previous studies [8,9,16]. The results demonstrate the competitive accuracy of our methods.

CIFAR: We further explore the performance of LDF-BNN versus advanced BNN (with ResNet-18 as the backbone) on the CIFAR10 and CIFAR100 small datasets in Table 4. Compared to BNext, LDF-BNN improved accuracy by 0.8% on CIFAR10 and 0.9% on CIFAR100.

#### 4.1.2. Ablation Experiments

We evaluated the effectiveness of the optimization scheme based on the experiment in Table 5. The experiment first removed the LDF part from LDF-BNN-S for training, and obtained a Top-1 accuracy of 75.27%. BNext-S improved the accuracy by 0.77% through its unique information recoupling structure, while LDF-BNN-S improved the accuracy by 0.48% by introducing LDF, which is close to the accuracy of SOTA BNext-S, demonstrating the effectiveness of LDF’s inter-layer information. Secondly, LDF-BNN-S was directly quantized to obtain an accuracy of 72.44%. The accuracy was reduced to 0.59% through modules merging, and the reduction was reduced to 0.21% by introducing the GAP factor, which fully demonstrated the effectiveness of the GAP factor in optimizing the accuracy of the quantized model.

#### 4.1.3. Performance on Mixed WM-38 Dataset

We apply different models to mixed-type wafer defect detection to further evaluate the performance in Table 6. On Mixed WM-38 [12] dataset, the LDF-BNN-S model approaching or exceeding 98.7%. The above illustrates that the unquantized LDF-BNN-S exceeds the accuracy standard of DNN in [2]. Quantization will have some impact on the performance of the model, but the accuracy drop will not exceed 1%. This shows that the quantized model still maintains a high accuracy. Meanwhile, the quantized LDF-BNN-S has significantly fewer parameters and lower computational complexity than traditional DNN ResNet-50, demonstrating advantages in parameter size, computational cost, and accuracy. Compared to the lightweight DNN MobileNetV2, the computational cost is reduced by 42.6%, and the accuracy is improved by 1.1%, although the number of parameters is increased by 27.8%. These advantages suggest that BNNs are a promising solution for industrial defect detection.

It is noteworthy that, in order to accommodate the LDF-BNN and to extract a richer set of features, the images in these datasets are also scaled to 224 × 224 × 3 size during the pre-processing stage. The Mixed WM-38 dataset contains a total of 38,015 images. Among them, there are 149 images in the “Near-Full” category and 866 images in the “Random” category. In order to balance the number of different categories, the “Near-Full” category and the “Random” category were increased to 1000 images by random flip and random rotation. Finally, the 39,000 images in the dataset were also divided into a training set and a test set in a ratio of 8:2.

### 4.2. Hardware Performance Analysis

**Figure 11:**
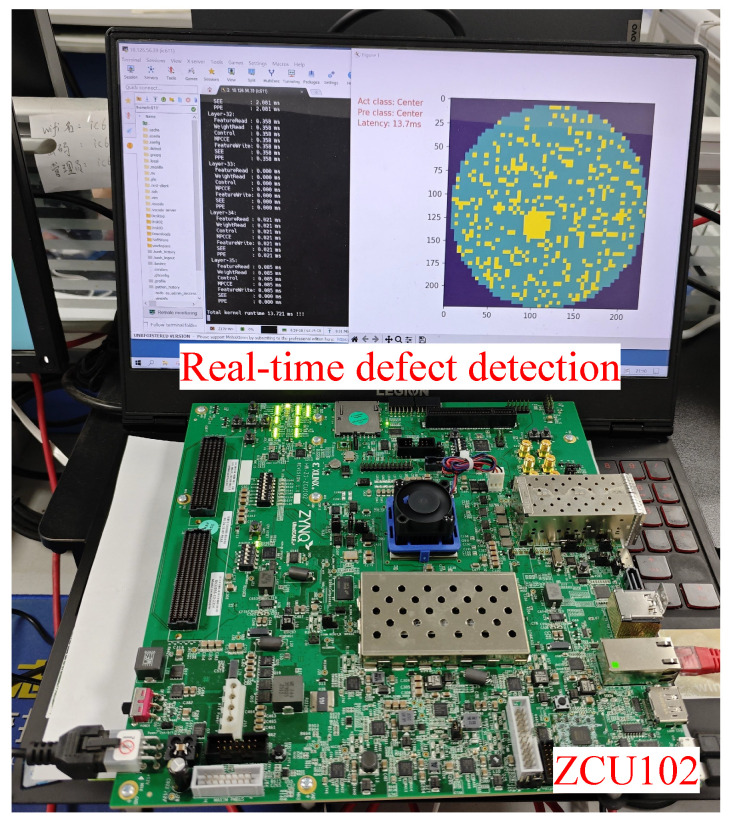
Running LDF-BNN on FPGA platform for defect detection. In the result image on the right, the purple represents the background, the green represents a normal grain, and the yellow represents a defective grain. The actual class and the predicted class are both center defect.

We implemented and deployed the flexible and configurable accelerator for the optimized BNext model on Zynq ZCU102. First, we used the Vitis HLS 2022.1 to convert MMCCEs, OCDBs, SEEs, and PPEs written in C++ into Register Transfer Level IP cores (RTL IP). Next, the RTL IP is imported into Vivado 2022.1, and control logic and memory controller are added to generate bitstream files. Finally, we use Vitis 2022.1 to compile the C++/OpenCL code to obtain the executable file, which implements the start/stop control of the kernel and the data interaction between the CPU and the FPGA. The specific classification results and performance obtained through inference on FPGA are shown in Figure 11.

#### 4.2.1. Comparison under Different Methods

We categorize the hardware architecture, which only adapts to BNext, as the baseline architecture. Simultaneously, we regard the hardware architecture, which accommodates LDF-BNN-S without MSP, as the optimized hardware architecture.

We have mapped the entire model on the ZCU102. In Table 7, we compare the performance and resource obtained by using different optimization methods. Baseline and optimized both use $N_{n}=3$, $N_{m}=16$, $N_{r}=14$ computing parallelism, and the method with MSP will increase the storage parallelism by $N_{o}=2$. In addition, the Conv1×1 and Conv3×3 under consideration correspond to the 8-bit convolution and 1-bit convolution of the blue convolution module depicted in Figure 1.

When compared with the baseline, the optimization has improved the average efficiency of the Conv1×1 by 14.27% and that of the Conv3×3 by 34.09%, which results in an overall speed-up factor of 1.83×. This indicates that the optimized architecture significantly improved the speed-up ratio, and is a noteworthy efficiency improvement over the baseline. As for hardware resources, it reduces 38.93% of DSP resources because its BN/Sigmoid are all optimized in the hardware. The 8-bit carry chain (CARRY8) and kLUTs are reduced due to the reduction of its logical calculations and multiplications. Additionally, the design of OCDB results in a 20.56% increase in BRAM usage, which is an acceptable trade-off for the achieved performance gains.

Further, when adding the MSP method to the optimized architecture, the average efficiency of the Conv1×1 is increased to 42.46%, and the average efficiency of Conv3×3 is increased to 93.73%, achieving a 2.21× speedup. Its latency is 13.7 ms, which indicates that it can effectively meet real-time requirements. Regarding hardware resources, it is found that the BRAM usage increases by 16 units due to the introduction of additional on-chip storage. Although this increases the complexity of scheduling computational tasks and results in an increase in the number of kLUTs, the increase in hardware resources is acceptable given the increased ability of the system to meet real-time performance requirements.

#### 4.2.2. Performance Comparison with Existing Designs

For fairness, we only compared networks implemented on the same hardware platform. We measured the total power consumption of the hardware platforms using a power meter and estimated the on-chip power consumption using the Vivado power report. In Table 8, we compared the implementations of different models on the ImageNet dataset on the ZCU102 FPGA. Notably, on the ImageNet dataset, our proposed LDF-BNN-S is the first model to achieve more than 72% accuracy while quantizing the activation of binary convolution to 1-bit. Meanwhile, the LDF-BNN-S achieves an impressive 72.6 FPS and 1826 GOPs, It surpasses the works cited in [10,18,19] with its high throughput. The theoretical throughput, calculated as $N_{m}\times N_{n}\times N_{r}\times F_{\max}\times 8$, is 2150.4 GOPs, confirming that our accelerator attains a total computing efficiency of 84.0%.

Regarding hardware resources, we evaluated the performance of different accelerators using GOPs/kLUT, GOPs/DSP and energy efficiency. Compared with the BNN accelerator VTA, our accelerator significantly surpasses it in both GOPs/kLUT and energy efficiency, and GOPs/DSP of our accelerator is also close to this index of it. Compared with the low-bit (2/4/5/8-bit) quantized DNN accelerators in [18,19], our accelerator shows remarkable advantages. Regarding power consumption, our accelerator consumes only 9.3 W, which is 27.9% lower than the 12.9 W of the FILM-QNN. Additionally, our accelerator has a higher GOPs/kLUT ratio of 10.49 compared to FILM-QNN’s 4.32/4.95/1.78 and LAMPS’s 3.67. Moreover, our energy efficiency of 196.3 GOPs/W beats FILM-QNN and LAMPS by at least 184.1%. Compared with high-precision (16-bit) DNN accelerators, as in [20,21], our accelerator has a certain gap in accuracy. However, it shows remarkable advantages in FPS and throughput. Specifically, FPS is increased by 548.2%, and throughput is increased by at least 527.4%. Moreover, energy efficiency is increased by at least 457.6%. In conclusion, the proposed accelerator outperforms previous studies in terms of GOPs/kLUT, GOPs/DSP, energy efficiency, and model accuracy balance.

#### 4.2.3. Performance Comparison with CPU and GPU

To benchmark our hardware accelerator against GPU and CPU, we implemented the LDF-BNN-S using the widely-adopted deep learning framework PyTorch version 1.13.1. The experimental setup encompassed an Intel i9-9900k desktop CPU and an NVIDIA Geforce RTX 4090 GPU. For equitable comparison, all tests were conducted with a batch size of one. Table 9 presents a comparative analysis of the performance for the three distinct implementations. The results show that while the model achieves the highest frame rate of 357.4 FPS on GPU, the FPGA-based accelerator achieves the highest energy efficiency of 7.81 FPS/W among the evaluated platforms.

## 5. Conclusions

In this paper, we propose an efficient accelerator based on LDF-BNN. The accelerator increases model performance through layered data fusion mechanism(LDF) and multi-storage parallelism(MSP). Ultimately, the proposed accelerator is implemented on a Xilinx ZCU102 FPGA, achieving an image processing performance of 72.23% high accuracy, 72.6 FPS and 1826 GOPs on the ImageNet dataset, which can effectively meet real-time requirements. And LDF-BNN can also achieve an accuracy of 98.70% on the Mixed WM-38 dataset, providing an effective solution for detecting defects in various industrial scenarios.

## Figures and Tables

**Table 1:** The configuration of LDF-BNN.

Models	N	M	Channel Number (C)
LDF-BNN-T	1	3	32
LDF-BNN-S	1	3	48

**Table 2:** Comparison of DRAM data transfer volume for the weights and I/OFMs of all layers.

Models	Weight	I/OFMs	Total
BNext-S	12.34 MB	58.85 MB	71.19 MB
LDF-BNN-S	12.34 MB	20.64 MB	32.98 MB
Reduction	0.00%	64.93%	53.67%

**Table 3:** Performance comparison with different BNNs on ImageNet.

Models	Year	BOPs ($10^{9}$)	Top-1 Accuracy (%)
Bi-RealNet-18 [8]	2020	1.68	56.4
Bi-RealNet-34 [8]	2020	3.53	62.2
ReActNet-A [9]	2020	4.82	69.4
ReActNet-Adam [16]	2019	4.82	70.5
BNext-T [7]	2022	4.82	72.4
BNext-S [7]	2022	10.84	76.1
LDF-BNN-T	2024	4.82	72.2
LDF-BNN-S	2024	10.84	75.8

**Table 4:** Performance comparison with different BNNs on CIFAR.

Models	Year	CIFAR10 Accuracy (%)	CIFAR100 Accuracy (%)
ReActNet [17]	2021	92.1	68.3
AdaBNN [17]	2022	93.1	-
BNext [7]	2022	93.6	72.2
LDF-BNN	2024	94.4	73.1

**Table 5:** Ablation experiments on different optimization strategies.

Methods	Epochs	Top-1 Accuracy (%)
Remove LDF	512	75.27
BNext-S	512	76.04 (+0.77)
LDF-BNN-S	512	75.78 (+0.48)
+quantize	128	72.44
+modules merging	128	71.85 (−0.59)
+GAP factor	128	72.23 (−0.21)

**Table 6:** Performance of different models on Mixed WM-38 dataset. The unit of the number of parameters in the table is megabyte (MB). The OPs are calculated from BOPs/64 + QOPs/8 + FLOPs, where FLOPs means floating point operations per second.

Models	Parameters (MB)	OPs (M)	Accuracy (%)
MobileNetV2 [2]	8.92	326.22	97.56
ResNet-50 [2]	94.08	4131.71	96.92
LDF-BNN-S	11.75	298.98	98.78
+quantize			
+modules merging			
+GAP factor	11.40	187.13	98.70

**Table 7:** Performance and resource comparison under different methods. Arrowrs represents the decrease/increase amount for the efficiency indicator, and represents the relative decrease/increase amount for other indicators.

Methods	Conv1×1 Efficiency	Conv3×3 Efficiency	Overall Efficiency	Latency (ms)	Speedup	kLUTs	BRAM	CARRY8	DSPs
Baseline	17.41%	46.70%	38.18%	30.3	1×	189	357.5	7979	863
optimized	31.68% (↑14.27%)	80.79% (↑34.09%)	69.70% (↑31.52%)	16.6	1.83×	171 (↓9.52%)	431 (↑20.56%)	6998 (↓12.29%)	527 (↓38.93%)
optimized+ MSP	42.46% (↑25.05%)	93.73% (↑47.03%)	84.45% (↑46.27%)	13.7	2.21×	174 (↓7.94%)	447 (↑25.03%)	7265 (↓8.95%)	527 (↓38.93%)

**Table 8:** Comparisons of different model implementations on the ImageNet dataset on ZCU102 FPGA. The power consumption indicated by “†” is obtained by both actual measurements and Vivado reports, where the actual measured power consumption is larger than the Vivado reported power consumption.

Accelerator	[20]	FILM-QNN [18]			HeatViT [21]	VTA [10]	LAMPS [19]	Ours
Year	2019	2022			2023	2024	2024	2024
Model	ResNet- 50	ResNet- 18	ResNet- 50	MobileNet- V2	DeiT-B	BiRealNet- 18	NAS VGG -16	LDF-BNN- S
Top-1 (%)	76.5	70.47	77.25	65.67	81.80	56.40	70.10	72.23
Bits (W/A)	16/16	4/5			16/16	1/1	Mixed	**1/1**
$F_{\max}$ (MHz)	200	150			150	333	214	200
Power †	23.6/-	-/12.9			-/11.0	-/-	-/-	18.6/9.3
kLUT	132	180			145	53	206	174
DSP	1144	2092			1786	59	1037	527
BRAM (36k)	912	440.5			664.5	139	481.5	447
FPS	-	214.8	109.1	537.9	11.2	24.3	40.6	72.6
Throughput (GOPs)	291	779	891	320	394	230	757	**1826**
GOPs/kLUT	2.20	4.32	4.95	1.78	2.72	4.34	3.67	**10.49**
GOPs/DSP	0.25	0.37	0.43	0.15	0.22	**3.89**	0.73	3.46
Energy efficiency † (GOPs/W)	12.33/-	-/60.4	-/69.1	-/24.8	-/35.2	-/-	-/-	**98.2/196.3**

**Table 9:** Performance comparison with CPU and GPU.

Platform	Frequency	Power (W)	FPS	Energy Efficiency (FPS/W)
CPU	3.6 GHz	95 W	2.3	0.02
GPU	2.2 MHz	302 W	357.4	1.18
FPGA	0.2 GHz	9.3 W	72.6	7.81

## Data Availability

The original contributions presented in the study are included in the article, further inquiries can be directed to the corresponding author.

## Nomenclature

The following nomenclature is used in this manuscript:

Conv2d	2D convolution
BConv	Binary convolution
BN	Batch normalization
PReLU	Parametric rectified linear unit
AvgPool	Average pooling
PWC	Point-wise convolution
GAP	Global average pooling
Move	Add the parameter in the per-channel direction in BNext
SE	Squeeze-and-excitation
B0	Block random access memory with id 0
MEM	Dynamic random access memory
CL3×3	Complex Layer with kernel size of 3 × 3
SL1×1	Simple Layers with kernel size of 1 × 1
2SL1×1	2 SL1×1 modules and concat
Conv3×3	Convolution with kernel size of 3 × 3
Conv1×1	Convolution with kernel size of 1 × 1
LDF-BNN-T	LDF-BNN with tiny model size
LDF-BNN-S	LDF-BNN with small model size
Accum	The accumulator
Reorder	Data reordering operation
*i*	A superscript means in the *i*-th layer
in	A subscript means the input of BConv
out	A subscript means the output of BConv
*W*	The width of feature map
*H*	The height of feature map
*C*	The number of channels in feature maps
*S*	The total size of the feature map

## Appendix A

In Table A1, we compare the performances of different activation methods in the LDF-BNN based on the ResNet-18 framework. On small datasets, ReLU has at least a 1.56% reduction in accuracy compared to Sigmoid. Moreover, the use of look-up tables by Sigmoid results in only a 0.58% increase in kLUT. In summary, the accuracy degradation after using ReLU is too severe to compare with the reference design, so we did not adopt this strategy.

**Table A1:** Performance comparison using different activation methods. Arrowrs represents the decrease/increase amount for the accuracy indicator, and represents the relative decrease/increase amount for other indicators.

Methods	CIFAR10 Accuracy	CIFAR100 Accuracy	kLUT
LDF-BNN(ResNet-18+ReLU)	92.83%	69.56%	173
LDF-BNN(ResNet-18+Sigmoid)	94.39% (↑1.56%)	73.07% (↑3.51%)	174 (↑0.58%)
